# Suppression of Hepcidin Expression and Iron Overload Mediate *Salmonella* Susceptibility in Ankyrin 1 ENU-Induced Mutant

**DOI:** 10.1371/journal.pone.0055331

**Published:** 2013-02-04

**Authors:** Kyoko E. Yuki, Megan M. Eva, Etienne Richer, Dudley Chung, Marilène Paquet, Mathieu Cellier, François Canonne-Hergaux, Sophie Vaulont, Silvia M. Vidal, Danielle Malo

**Affiliations:** 1 Department of Human Genetics, McGill University, Montréal, Quebec, Canada; 2 Department of Medicine, McGill University, Montréal, Quebec, Canada; 3 Complex Traits Group of the McGill Life Sciences Complex, McGill University, Montréal, Quebec, Canada; 4 Comparative Medicine and Animal Resources Centre, McGill University, Montréal, Quebec, Canada; 5 Department of Biochemistry and Microbiology, University of Victoria, Victoria, British Columbia, Canada; 6 Centre INRS-Institut Armand Frappier, Laval, Quebec, Canada; 7 INSERM U1043-CPTP, Toulouse, France; 8 INSERM, U1016, Institut Cochin, Paris, France; 9 CNRS, U5282, Toulouse, France; 10 Université de Toulouse, UPS, Centre de Physiopathologie de Toulouse Purpan (CPTP), Toulouse, France; Indian Institute of Science, India

## Abstract

*Salmonella*, a ubiquitous Gram-negative intracellular bacterium, is a food borne pathogen that infects a broad range of hosts. Infection with *Salmonella* Typhimurium in mice is a broadly recognized experimental model resembling typhoid fever in humans. Using a *N*-ethyl-*N*-nitrosurea (ENU) mutagenesis recessive screen, we report the identification of *Ity16* (Immunity to Typhimurium locus 16), a locus responsible for increased susceptibility to infection. The position of *Ity16* was refined on chromosome 8 and a nonsense mutation was identified in the ankyrin 1 (*Ank1*) gene. ANK1 plays an important role in the formation and stabilization of the red cell cytoskeleton. The *Ank1^Ity16/Ity16^* mutation causes severe hemolytic anemia in uninfected mice resulting in splenomegaly, hyperbilirubinemia, jaundice, extramedullary erythropoiesis and iron overload in liver and kidneys. *Ank1^Ity16/Ity16^* mutant mice demonstrated low levels of hepcidin (*Hamp*) expression and significant increases in the expression of the growth differentiation factor 15 (*Gdf15*), erythropoietin (*Epo*) and heme oxygenase 1 (*Hmox1*) exacerbating extramedullary erythropoiesis, tissue iron deposition and splenomegaly. As the infection progresses in *Ank1^Ity16/Ity16^*, the anemia worsens and bacterial load were high in liver and kidneys compared to wild type mice. Heterozygous *Ank1^+/Ity16^* mice were also more susceptible to *Salmonella* infection although to a lesser extent than *Ank1^Ity16/Ity16^* and they did not inherently present anemia and splenomegaly. During infection, iron accumulated in the kidneys of *Ank1^+/Ity16^* mice where bacterial loads were high compared to littermate controls. The critical role of HAMP in the host response to *Salmonella* infection was validated by showing increased susceptibility to infection in *Hamp*-deficient mice and significant survival benefits in *Ank1* ***^+/Ity16^*** heterozygous mice treated with HAMP peptide. This study illustrates that the regulation of *Hamp* and iron balance are crucial in the host response to *Salmonella* infection in *Ank1* mutants.

## Introduction

*Salmonella* infections in humans are responsible for two major diseases, typhoid fever caused by *Salmonella* Typhi and *Salmonella* Paratyphi and a diarrheal disease known as salmonellosis caused by several non host specific serotypes including *Salmonella* Typhimurium and *Salmonella* Enteritidis. Typhoid is transmitted by a fecal-oral route through contaminated food and water and is endemic in areas of poor water sanitation. It is estimated by the World Health Organization that there are 21 million new cases of typhoid fever each year resulting in approximately 200,000 deaths. In addition, 1 to 5% of patients become asymptomatic chronic carriers serving as reservoirs from which new *Salmonella* Typhi infections can be transmitted [1]. Early during infection, phagocytes are instrumental in the control of bacterial replication through the production of pro-inflammatory cytokines such as IFNγ, TNFα and IL-12. Immunocompromised patients due to chronic granulomatous disease (deficient in NADPH oxidase activity), defects in the IFNγ or IL-12 signaling pathway or HIV infection are more susceptible to disseminated *Salmonella* infection [2], [3], [4]. In addition, patients with hemoglobinopathies resulting in iron overload such as sickle cell anemia and thalassemia present increased susceptibility to *Salmonella* infection [5], [6]. In the mouse, *Salmonella* Typhimurium infection results in a systemic disease with clinical manifestations resembling those found in humans with typhoid fever. The severity of infection depends on a variety of factors including the dose of the inoculum, as well as the interaction of host and bacterial genetic determinants. Several strains of mice show varying degrees of susceptibility to *Salmonella* Typhimurium infection with the mouse strain 129 substrains showing the most resistance. The study of the natural variation of the host response to infection with *Salmonella* Typhimurium in spontaneous mouse mutants have identified important innate immune genes having Mendelian contribution to disease susceptibility and contributing to different mechanisms including pathogen recognition (Tlr4^P712H^ in C3H/HeJ), phagosome transport of divalent cations including iron (Nramp1*^G169D^* in C57BL/6J and BALB/cJ) or erythropoiesis and iron metabolism (Pklr^I90N^ in AcB61 mice) [7],[8],[9],[10].

Due to the limited amount of natural variation found in classical inbred mice, we have used *N*-ethyl-*N*-nitrosurea (ENU) mutagenesis to generate novel mutations responsible for increased susceptibility to *Salmonella* infection. Recessive ENU induced mutations are bred to homozygosity with the use of a three-generation breeding scheme and challenged with *Salmonella* Typhimurium. In the current screen we have evaluated 216 pedigrees for their susceptibility to infection and identified 5 deviant pedigrees. In this paper, we report the identification of one of these ENU mutants named *Ity16* that carries a nonsense mutation in the gene *Ank1*. *Ank1* encodes a red blood cell (RBC) adaptor protein consisting of three major functional domains: an N-terminal membrane binding, a spectrin binding and a C-terminal regulatory domain [11]. ANK1 plays an important role in RBC membrane stability by mediating the attachment of band 3 (SLC4A1) and protein 4.2 (EPB4.2) to the spectrin-based membrane cytoskeleton [12]. A number of mutations in murine *Ank1*, both spontaneous and ENU-induced, have been described to cause hemolytic anemia [13], [14], [15], [16], [17]. In humans, mutations within ANK1 cause hereditary spherocytosis, a disease characterized by hemolytic anemia [18], [19], [20]. In the current paper, we show that *Ank1^Ity16/Ity16^* and *Ank1^+/Ity16^* mice have increased susceptibility to *Salmonella* Typhimurium infection, although in the latter group the increased susceptibility is delayed and milder. The suppression of hepcidin (*Hamp*) expression and iron overload contribute to their increased susceptibility to *Salmonella* infection.

## Materials and Methods

### Mice and ENU Mutagenesis

All animal experiments were performed under conditions specified by the Canadian Council on Animal Care and the animal use protocol was approved by McGill University Facility Animal Care Committee. Generation 0 (G0) 129S1 males were mutagenized with a single injection of 150 mg per kg of body weight of *N*-ethyl-*N*-nitrosurea (ENU) given intraperitoneally. G0 males were outcrossed to 129X1 females to create G1 progeny that were subsequently crossed to DBA/2J. Resulting G2 animals were intercrossed to produce the G3 mice that were phenotyped for their susceptibility to *Salmonella* infection. Hamp^tm1Svl^ knock out mice [21] were transferred onto a 129S6 background, a strain that carries a wild-type allele of *Slc11a1* (129S6.B6*129S2-Hamp^tm1Svl^).

### Genotyping

The genome scan was performed using a 1441 SNP panel (The Centre for Applied Genomics, Toronto, Ontario, Canada) on 22 mice. Additional genotyping was performed by microsatellite analysis or restriction enzyme digests of SNP markers. Sequencing of the Ank1 gene was performed on cDNA isolated from spleens of *Ank1^ty16/Ity16^* and *Ank1^+/+^* mice using 3730xl DNA Analyzer (Applied Biosystems) from the McGill University and Genome Quebec Innovation Centre.

### In vivo *Salmonella* Infections

Mice were infected with 5000 CFUs of *Salmonella* Typhimurium strain Keller as described by us previously [22]. The infectious inoculum was diluted to 25,000 CFUs per mL and 0.2 mL was injected into the caudal vein of 7 week old mice of both sexes. To determine CFUs in tissues, mice were euthanized with CO_2_ at day 2 and day 6 post infection and spleen, liver and kidney were removed aseptically, weighed and homogenized. Homogenates were diluted in saline and plated on trypticase soy agar (TSA) overnight. Other groups of mice were coinfected with 2500 CFUs of *Salmonella* Typhimurium strain Keller and 2500 CFUs of Δ*tonB* constructed by allelic exchange in serovar Keller [23]. The tissues were collected 2 days after infection and tissue homogenates were plated on TSA containing or not 50 mg/ml of kanamycin.

### Western Blots

Erythrocyte ghost membranes were prepared by osmotic lysis as previously described ([15]). Primary antibody to detect ANK1 (N13) was purchased from Santa Cruz Biotechnology (SC-87552) and was used at a 1∶200 dilution. This antibody was raised against a peptide sequence spanning exons 8 and 9 of the *Ank1* transcript (ENSMUST00000121802) and recognized the full length ANK1. The ACTIN antibody and the secondary antibodies for Western detection (anti-rabbit IgG) were purchased from Cell Signaling Technology.

### Hepcidin Treatment

Mice were treated with 50 µg of HAMP (Peptide International, Louisville KY) resuspended in 100 µl of PBS intraperitoneally. Four hours later, mice were infected with 5000 CFUs of *Salmonella* Typhimurium and monitored for survival over a period of 14 days. Littermate controls were given 100 µl of PBS intraperitoneally.

### Histology

Tissues were collected from *Ank1^Ity16/Ity16^*, *Ank1^+/Ity16^* and *Ank1^+/+^* mice and fixed in 10% neutral buffered formalin for 24 hours at 20°C, then placed in 70% ethanol at 4°C before processing and embedding (Goodman Cancer Research Center histology facility, McGill University). Embedded tissues were sectioned and stained with hematoxylin and eosin or prussian blue.

### Hematologic and Biochemical Parameters

Blood samples from *Ank1^Ity16/Ity16^*, *Ank1^+/Ity16^* and *Ank1^+/+^* mice were collected by cardiac puncture and analyzed for CBC & differential and reticulocyte counts (Diagnostic Research Support Service, Comparative Medicine and Animal Resources Centre, McGill University). Serum was isolated with a serum separator tube (Sarstedt) and analyzed for bilirubin, blood urea nitrogen (BUN), alanine transaminase (ALT) and aspartate transaminase (AST) (Diagnostic Research Support Service, Animal Resources Centre, McGill University).

### QRT-PCR

Total RNA was extracted from spleen, liver and kidneys with Trizol Reagent (InVitrogen, Burlington ON) according to manufacturer instructions. cDNAs were synthesized using SuperScript®II Reverse Transcriptase (InVitrogen). Quantitative PCR was performed on a Chromo4 (BioRad, Mississauga ON) or StepOnePlus (Applied Biosystem, Carlsbad CA) using SYBR Green (Applied Biosystems) for hepcidin (*Hamp*), ferroportin (*Slc40a1*), *Il6*, *Il1*, *Ifng*, erythropoietin (*Epo*), heme oxygenase 1 (*Hmox1*) and growth differentiation factor 15 (*Gdf15*) expression. Two housekeeping genes: *Tbp* (TATA box binding protein) and *Hprt* (hypoxanthine guanine phosphoribosyl transferase) were used. The relative expression of the genes was normalized to the amount of *Tbp* and *Hprt* (endogenous reference) and relative to a calibrator (untreated wild type genotype) for each tissue by using the comparative 2^(−Delta Delta Ct)^ method. The primer sequences are provided in Table S1.

### Statistical Analysis

Data (unless otherwise specified) was analyzed by two-tailed Mann Whitney test and two-way ANOVA using GraphPad Prism 5. Data for qPCR was analyzed in R by ANOVA and Welch two sample t-test [24].

## Results

### Identification of a Novel *Salmonella* Susceptibility Locus using ENU Chemical Mutagenesis

**Figure 1:**
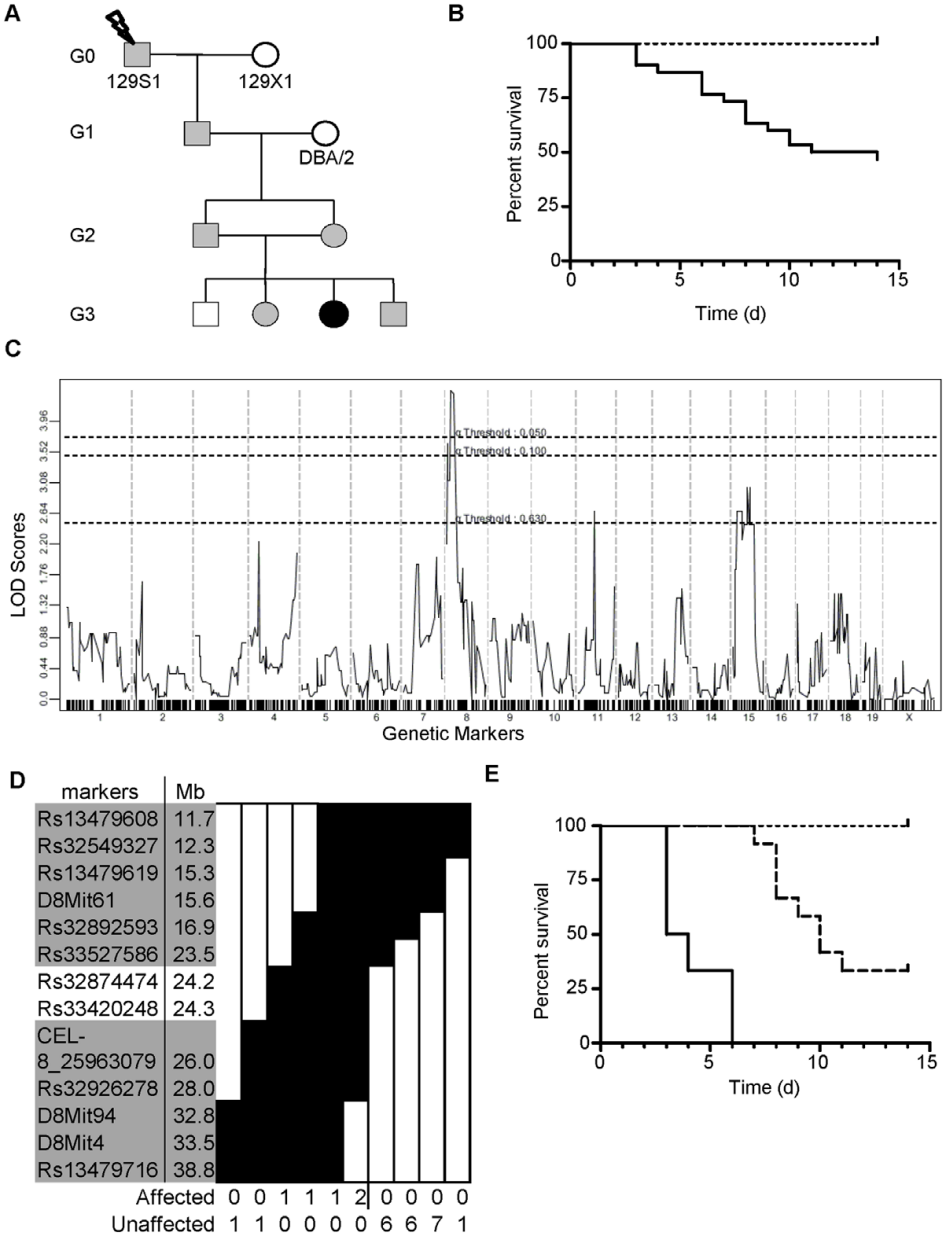
Identification and mapping of the *Ity16* pedigree. A) Breeding scheme used to identify and map the mutant family. Females and males are represented by circles and squares respectively with black representing the homozygous mutant alleles, gray representing the heterozygous alleles, and white representing the wild type alleles. B) Survival curve of the G3 mice from the *Ity16* pedigree is represented by the solid line (n = 30) while wild type 129S1 (n = 5) mice are represented by dotted lines. Log-Rank (Mantel-Cox) p = 0.01. C) Linkage analysis in 8 *Salmonella*-susceptible and 14 *Salmonella*-resistant mice identifies a significant peak on chromosome 8. D) Fine mapping of the *Ity16* locus to a 2.5 Mb region on chromosome 8. Black fill represents homozygous 129S1 allele. White fill represents heterozygous or homozygous DBA/2 genotypes. E) Survival curves of *Ity16* mice according to their genotypes at peak marker on chromosome 8. Solid line represents homozygous 129S1 alleles (n = 6), dashed line represents heterozygous (n = 12), and dotted line represents homozygous DBA/2 alleles (n = 12). Log-Rank (Mantel-Cox) for *Ank1^+/+^* and *Ank1^Ity16/Ity16^* p<0.0001, *Ank1^+/+^* and *Ank1^+/Ity16^* p = 0.001.

The breeding scheme used to identify the *Ity16* pedigree is shown in Fig. 1A. Mutagenized 129S1 G0 males were crossed to wild-type 129X1 females to produce G1 males. These G1 males were crossed to DBA/2J females to generate G2 offspring that were randomly intercrossed to produce G3 progeny. G3 animals were phenotyped for susceptibility to *Salmonella* infection with 5,000 CFUs and monitored for a period of 14 days. Mortality in the *Ity16* pedigree was observed between day 3 post infection and continued until day 11 with 25% of mice succumbing to infection by day 6 (Fig. 1B). An initial genome scan was performed using twenty-two G3 animals (8 susceptible and 14 resistant mice) and 708 informative DNA markers. Binary analysis of the genome scan revealed a locus on chromosome 8 at position 25.8 Mb with a LOD score of 3.72 (Fig. 1C). DNA samples used in the genome scan were also genotyped with DNA markers discriminating 129S1 and 129X1 genomes to ensure that mice were homozygous for the mutagenized 129 allele (data not shown). Fine mapping of the chromosome 8 region with 27 additional G3 animals and 9 markers reduced the region to a 2.5 Mb region (Fig. 1D) that includes 23 annotated genes, 10 predicted genes and 2 miRNAs (MGI). The survival curves of the *Ity16* G3 mice were plotted according to their genotype at the peak marker (rs32874474) on chromosome 8 (Fig. 1E). All mice homozygous for the 129S1 allele succumbed to infection by day 6. Mice carrying the DBA/2J alleles at this locus were 100% resistant to infection. Interestingly, mice with a heterozygous genotype at the peak marker had an intermediate survival phenotype, with mortality starting at day 7 (Fig. 1E).

### *Ity16* Mutant Mice Present Severe Hemolytic Anemia that Worsened during Infection

**Figure 2:**
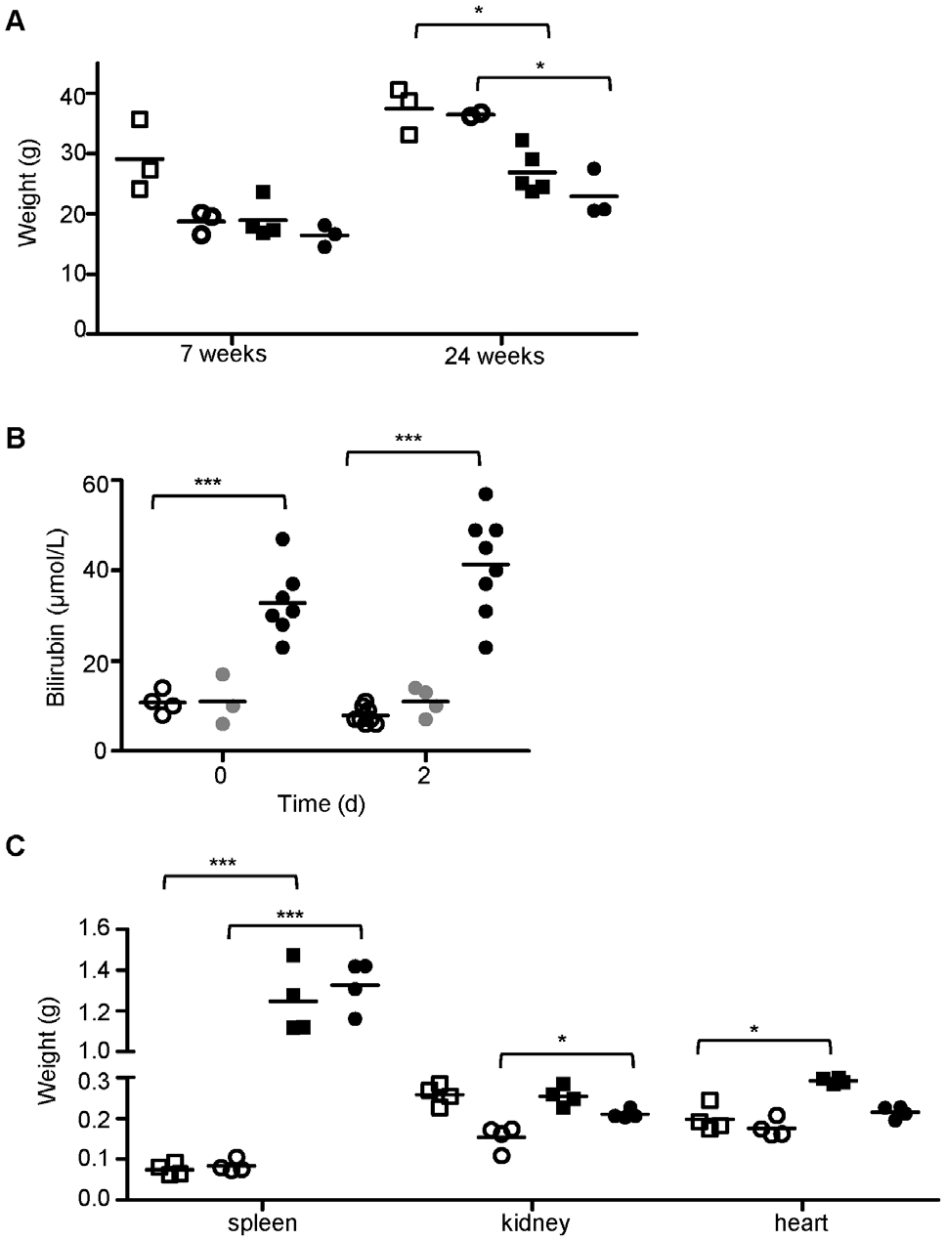
*Ity16* mutant mice present massive splenomegaly, moderate increase in kidneys and heart weights and bilirubinemia. A) Body weight of wild type littermates and *Ity16* mice by sex (males are represented by squares, females by circles) at 7 weeks (open and black fill) and 24 weeks (open and black fill) n = 3 per sex and genotype. B) Serum bilirubin levels were measured at day 0 and day 2 post infection in wild type (open circle), heterozygous (gray circle) and *Ity16* mutant (black circle) mice. C) Increased spleen, kidney and heart weights in *Ity16* mutant (black fill) mice compared to wild type littermates (open fill). The data are presented by sex (males are squares, females are circles). Results are representative of at least two experiments. An * represents a P-value of less than 0.05; ** represents a P-value of less than 0.001; *** represents a P-value of less than 0.0001.

Compared to wild type and heterozygous littermates, mutant mice are characterized clinically by low body weight (Fig. 2A), pallor of mucosal linings and yellow discoloration of subcutaneous tissues, a consequence of increased levels of bile pigment (bilirubin) in the blood (Fig. 2B). In addition, these mutants exhibit impressive enlargement of the spleen, accounting for up to 10% of their body weight (Fig. 2C). This is accompanied by significant increases in kidney and heart weights (Fig. 2C). Hematological analysis of uninfected mutant *Ity16* mice showed they have severe constitutive anemia as demonstrated by low hematocrit levels (25% in mutants compared to 50% in wildtype and heterozygous mice) which decreased to as low as 15% 2 days after infection (Table 1). This decrease in hematocrit levels is mirrored by similar decreases in hemoglobin and red blood cell levels during infection (Table 1). Additionally, there were a higher percentage of circulating reticulocytes in the blood of mutants compared to both wild type and heterozygous littermates (Table 1). As a consequence of severe anemia, *Ity16* mutant mice present extensive extramedullary erythropoiesis in the spleen and liver as assessed by histopathological examination that is associated in the spleen with severe lymphoid depletion (Fig. S1). Blood smear examination of mutant *Ity16* mice showed abnormally shaped red blood cells, marked anisocytosis, and reticulocytosis and the presence of spherocytes (data not shown). Total number of WBC, neutrophils and lymphocytes were significantly higher in *Ity16* mutant mice compared to littermate controls before and after infection (Table 1) after correcting for the high percentage of nucleated RBC (Table 1). The total number of neutrophils increased significantly during infection in all three groups although it was more pronounced in *Ity16* mutants.

**Table 1:** Hematologic parameters in *Ity16* mutant, heterozygous and wild type mice before (day 0) and after (day 2) infection with *Salmonella* Typhimurium.

	Day 0			Day 2		
Genotype	Wt	Het	*Ity16*	Wt	Het	*Ity16*
Hematocrit L/L	0.523±0.007	0.549±0.017	0.252±0.013*	0.519±0.011	0.526±0.023	0.190±0 020*
Hemoglobin g/L	167±0.88	169±5.57	76.9±4.02*	164±3.26	165±7.22	57.5±6.17*
RBCs×10^12^/L	11.5±0.39	13.2±0.21	5.47±0.25*	10.7±0.23	11.9±0.48	3.98±0.39* #
WBCs×10^9^/L	10.76±2.92	9.73±1.64	35±5.07	9.85±0.65	11.13±1.73	22.92±3.01*
% reticulocytes	5.1±0.17	5.03±0.64	44.9±7.68*	4.7±0.42	5.1±1.36	33.1±5.60*
neutrophils×10^9^/L	1.17±0.38	0.86±0.12	2.36±0.99	2.08±0.25	2.55±0.38	7.54±0.98*
lymphocytes×10^9^/L	9.54±2.6	8.82±1.57	32.24±4.59*	7.54±0.52	8.54±1.50	14.88±2.71*
Nucleated RBCs/100 WBCs	0	2.33±1.20	66.86±11.15*	3.38±1.74	3±3	77±5.58*

Wt : wild-type; Het : heterozygous.

* p values <0.05 compared to wild type values of the same infection status.

# p values <0.05 compared to day 0 of the same genotype.

### A Mutation within *Ank1* is Responsible for Susceptibility of *Ity16* Mice to *Salmonella* Infection

**Figure 3:**
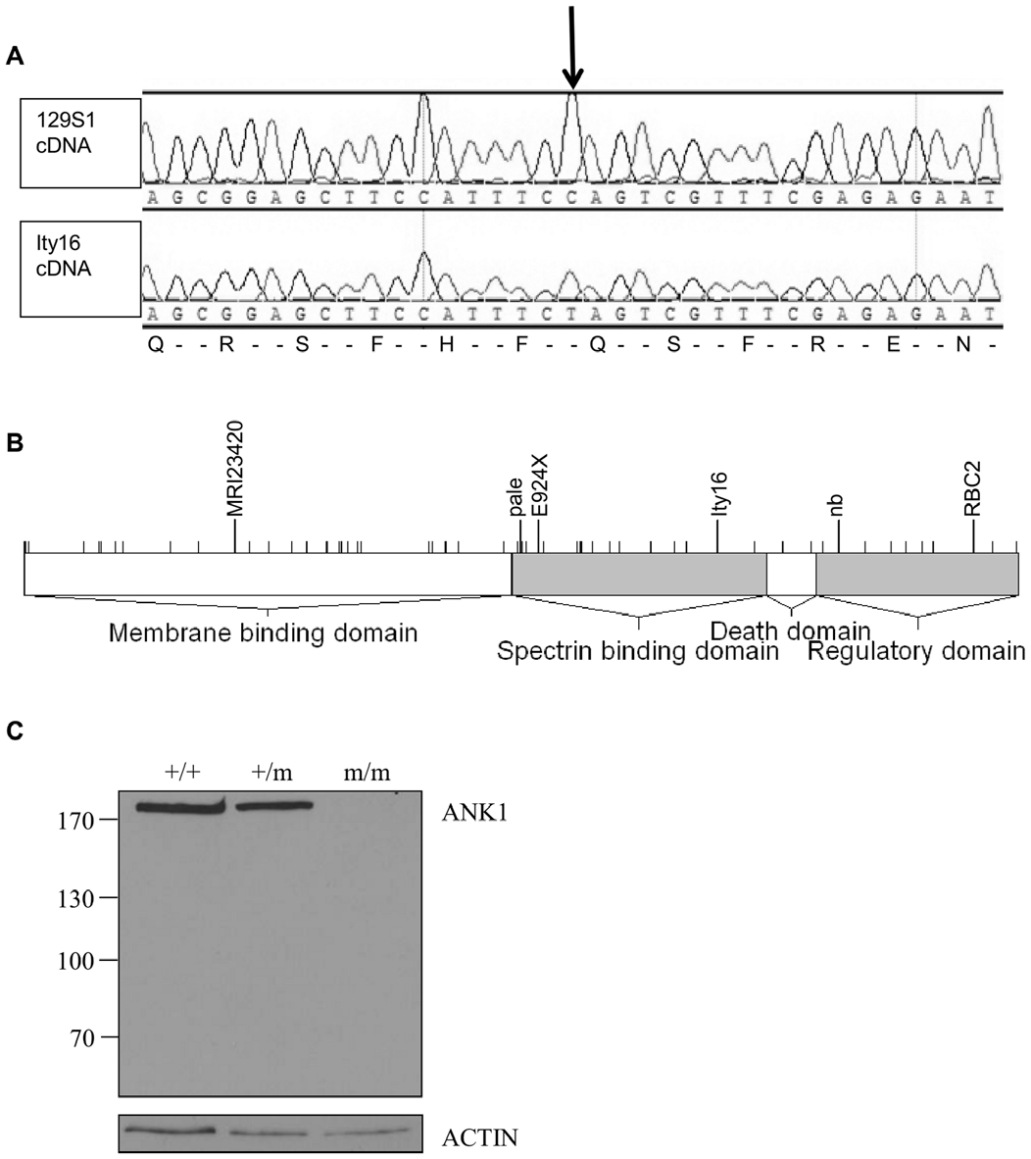
*Ity16* mutant mice carry mutation within the *Ank1* gene. A) A C to T transition (arrow) at cDNA position 4069 (c.4069C>T) resulting in a stop codon at amino acid position 1357 (p.Gln1357Ter) was detected in *Ank1^Ity16/Ity16^* mutant. The *Ity16* mutation is located in exon 33 using the exon numbering of the *Ank1* transcript ID ENSMUST00000121802 (Ensembl build 37). B) Schematic representation of the different domains of ANK1 (adapted from [57]) and location of mutations previously identified in human (small ticks) [18] and mouse ANK1 [13], [14], [15], [16], [17]. The *Ity16* allele is shown together with two spontaneous ANK1 recessive mutations (*nb and pale*) and three dominant mutants (*RBC2*, *M1Wlst* and *MRI23420*). C) Immunoblot of RBC ghosts prepared from wild-type (+/+), heterozygous (+/m) and mutant Ank1 (m/m) littermates probes with ANK1 antibody. The size of the molecular markers in kDa are shown on the left. An expected band of about 210 kDa was observed in samples from the wild-type and heterozygous mice while this band or any smaller bands corresponding to a truncated version of the protein could not be detected in mutant mice.

Identification of the causative gene for *Ity16* focused on the region between 23.5 Mb and 26 Mb on mouse chromosome 8. Genes involved in hematopoiesis and causing splenomegaly and anemia were of particular interest because of the distinct phenotype exhibited by the mutant mice. Only the gene ankyrin 1 (*Ank1*) met our criteria. ANK1 is primarily known for its structural role in erythrocytes. *Ank1* is part of a small gene family which members are adaptor structural components linking lipid membranes to the cytoskeleton showing an essential role in the stability of plasma membranes of many cell types [25]. The *Ank1* gene comprises a total of 44 exons. Several *Ank1* splice variants are observed and the full-length isoform encodes for a protein of 1907 amino acids (200−210 kDa). Erythroid *Ank1* consists of three major conserved domains including an N-terminal membrane-binding domain, a spectrin-binding domain and a C-terminal regulatory domain containing a death domain motif [26], [27]. *Ity16* mutant showed a C to T transition at cDNA position 4069 (c.4069C>T) resulting in a nonsense mutation in exon 33 at amino acid position 1357 (p.Gln1357Ter ) (Fig. 3A). This pre-mature stop codon results in a predicted truncation of the ANK1 protein by 550 amino acids with the loss of the C-terminal regulatory and the death domains but retaining the membrane-binding and most of the spectrin-binding domains (Fig. 3B). Immunoblotting analysis of fractioned erythrocyte membrane ghosts revealed that the non-sense mutation abrogated ANK1 protein expression in *Ity16* mutant mice (Fig. 3C). A truncated form of the protein (calculated molecular weigth of 144 kDa) could not be detected suggesting that the abnormal protein is targeted for degradation. The frequency of adult mice (older than 4 weeks) on a 129S1/DBA2J hybrid background with a c.4069C>T genotype was not in Hardy-Weinberg equilibrium: we obtained a ratio that was significantly different (p = .04) from the expected 1-2-1 ratio for a cross between mice heterozygous (+/m) for the mutation. Genotype frequencies in 249 animals were 29% for wild type mice, 52% for heterozygous and 18% for homozygous mutants.

### Iron Overload in *Ank1^Ity16/Ity16^* Mutant Mice Facilitates Proliferation of *Salmonella* in Liver and Kidneys

**Figure 4:**
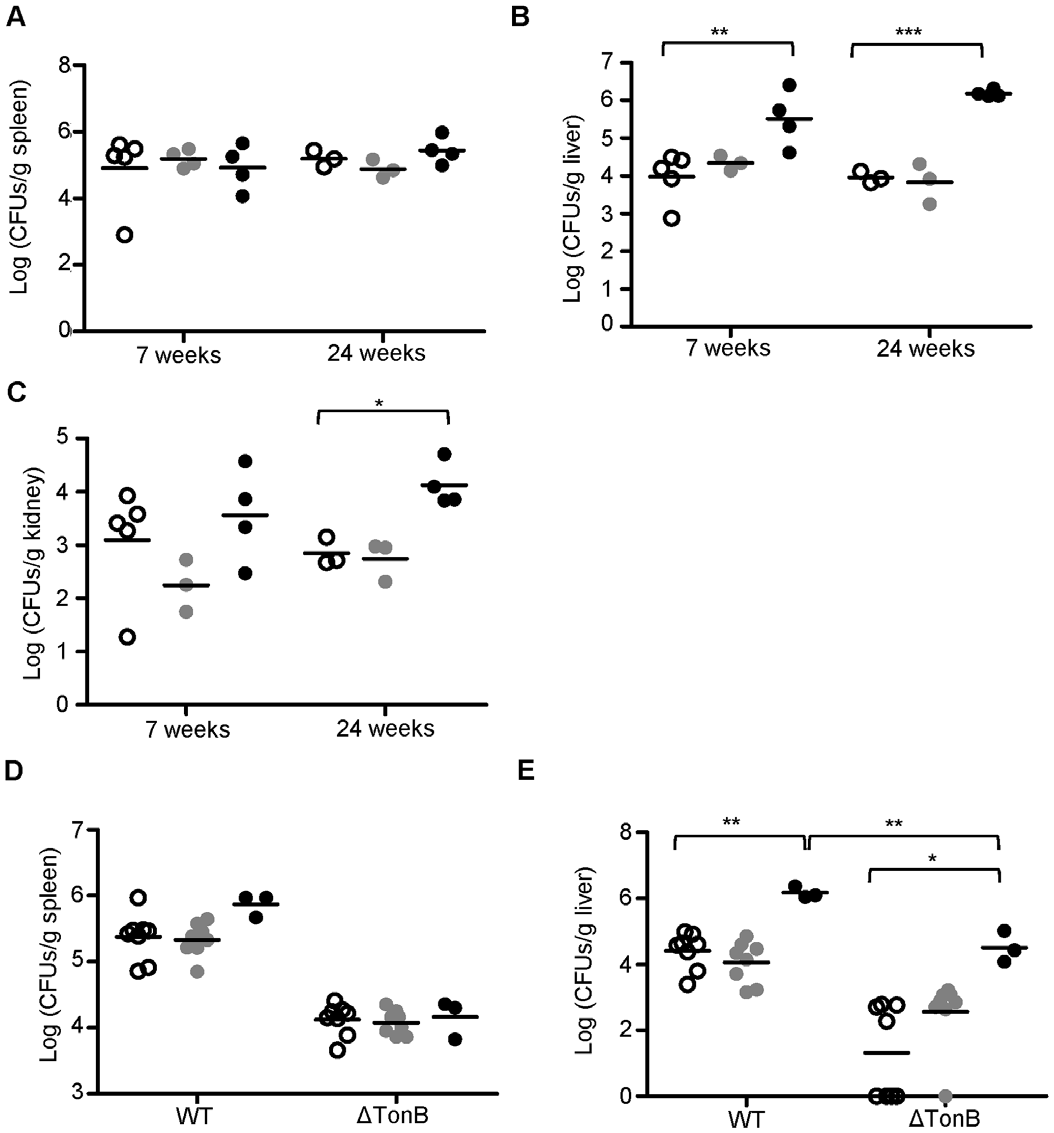
Bacterial load in spleen, liver and kidney after infection with *Salmonella* Typhimurium in *Ity16* mutant, heterozygous and wild type mice. The bacterial load was measured 2 days after infection in the spleen (A), liver (B), and kidney (C) of wild type aged 7 weeks (open circle), and 24 weeks (open circle), heterozygous mice at 7 weeks (gray circle) and 24 weeks (gray circle) and *Ity16* mutant mice at 7 weeks (filled circle) and 24 weeks (filled circle) (n = 3–5 per genotype and age). *Ity16* mutant mice present higher bacterial load in the liver and kidneys compared to heterozygous and wild type littermates. Bacterial load of *Salmonella* Typhimurium and *ΔtonB Salmonella* Typhimurium was measured 2 days after infection in spleen (D) and liver (E) of wild type (open circle), heterozygous (gray circle) and *Ity16* (black circle) mice aged 7–12 weeks (n = 3–8 per genotype). Results are representative of at least two experiments. An * represents a P-value of less 0.05; **represents a P-value of less than 0.001; ***represents a P-value of less than 0.0001.

**Figure 5:**
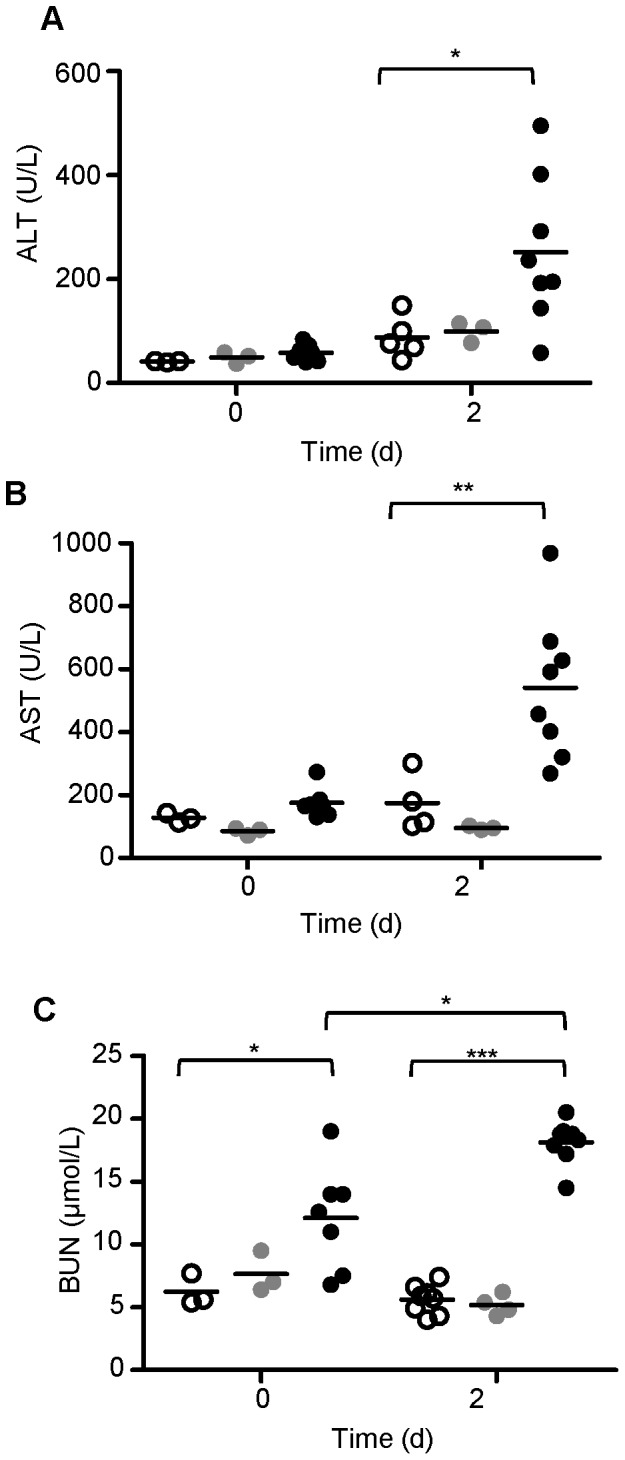
*Ity16* mutant mice present elevated blood urea nitrogen (BUN), alanine transaminase (ALT) and asparatate transaminase (AST) during *Salmonella* infection. BUN (A), ALT (B) and AST (B) levels were measured at day 0 (prior to infection) and day 2 post infection in 7 week old *Ank1^+/+^* wild type, *Ank1^+/Ity16^* heterozygous and *Ank1^Ity16/Ity16^* mutant mice. The values for each individual mouse are shown (n = 3–8 per genotype). Wild type mice are represented by open circles, heterozygous by gray circles and *Ity16* mutant by black circles. The high levels of BUN levels detected in mutant mice at day 0 most probably reflect the nephrotoxicity of iron accumulation in the kidney. During infection BUN, ALT and AST levels increased in mutant mice denoting kidney and liver damage. An *represents a P-value of less than 0.05; *** represents a P-value of less than 0.0001.

Increased amounts of iron pigment deposition were detected in *Ank1^Ity16/Ity16^* mutant livers and kidneys (Prussian blue positive pigment) compared with wild type and heterozygous littermates in non-infected conditions and in mice at 2 days after infection (Fig. S1). Iron accumulates predominantly in the hepatocytes and in the cytoplasm of the epithelium of the renal tubules. The high levels of iron in the liver and kidneys of *Ank1^Ity16/Ity16^* mutant mice suggest that the rapid turnover of RBC in these mice leads to accumulation of iron in the liver and kidneys (secondary hemochromatosis). In contrast, splenic iron content was markedly decreased in *Ank1^Ity16/Ity16^* mutant mice compared to wild type littermates (Fig. S1). In *Ank1^Ity16/Ity16^* mutant mice, iron overload is associated with increased bacterial proliferation in the liver and kidneys. In infected mice, there was a gradation of microabcesses present in the liver that worsened with genotype but was not influenced by the age at the time of infection (Fig. S2). In addition, significant higher bacterial loads were observed in the liver (30–100 times higher) and kidneys (10 times higher) of *Ank1^Ity16/Ity16^* mutant compared to wild type and heterozygous littermates 2 days post infection (Fig. 4B and 4C). Because of the high bacterial burden present in the liver and kidney, biochemical measures of hepatic (alanine transaminase, ALT and aspartate transaminase, AST) and kidney function (blood urea nitrogen, BUN) were evaluated before and after infection. ALT and AST were elevated after infection only in *Ank1^Ity16/Ity16^* mutant mice (Fig. 5A and 5B) whereas BUN levels were significantly higher in uninfected and infected *Ank1^Ity16/Ity16^* mice compared to controls (Fig. 5C). These data may indicate the presence of abnormal organ function in naïve *Ank1^Ity16/Ity16^* mice (kidney) and during infection (liver and kidney). In fact, we observed glomeruli with enlarged mesangium in *Ank1^Ity16/Ity16^*mice, a pathological change that may be compatible with membranoproliferative glomerulonephritis (Fig. S2).

To examine if iron accumulation in tissues has an impact on the bacterial load phenotype in *Ank1^Ity16/Ity16^* mutant mice, we infected mice with Δ*tonB Salmonella* Typhimurium. The *tonB* mutation is known to inactivate several siderophore-dependent Fe^3+^ uptake systems in *Salmonella* and Δ*tonB Salmonella* are as virulent as the wild-type strain in *Slc11a1*-deficient mice [23]. In the spleen where there is no iron accumulation, there was no significant genotype difference for the proliferation of Δ*tonB* and wild-type *Salmonella* (Fig. 4D). In contrast, the levels of both Δ*tonB* and wild-type bacteria were significantly higher (by a factor of ∼2 Log) in the liver of mutant mice where there is iron overload (Fig. 4E). At this early time point, there was no iron accumulation in the liver of heterozygous mice (Fig. S1) and the mice were able to control bacterial growth as well as wild-type mice (Fig. 4E). The growth of Δ*tonB Salmonella* appears to be affected by the presence of iron in the cellular microenvironment, suggesting that iron uptake via tonB is not limiting when iron is abundant in tissue. Overall, these data support the observation that tissue iron overload in *Ank1^Ity16/Ity16^* mutant promotes *Salmonella* growth.

### Increased Expression of *Epo* and *Gdf15* Paralleled Low Levels of *Hamp* Expression in *Ank1^Ity16/Ity16^* Mice

**Figure 6:**
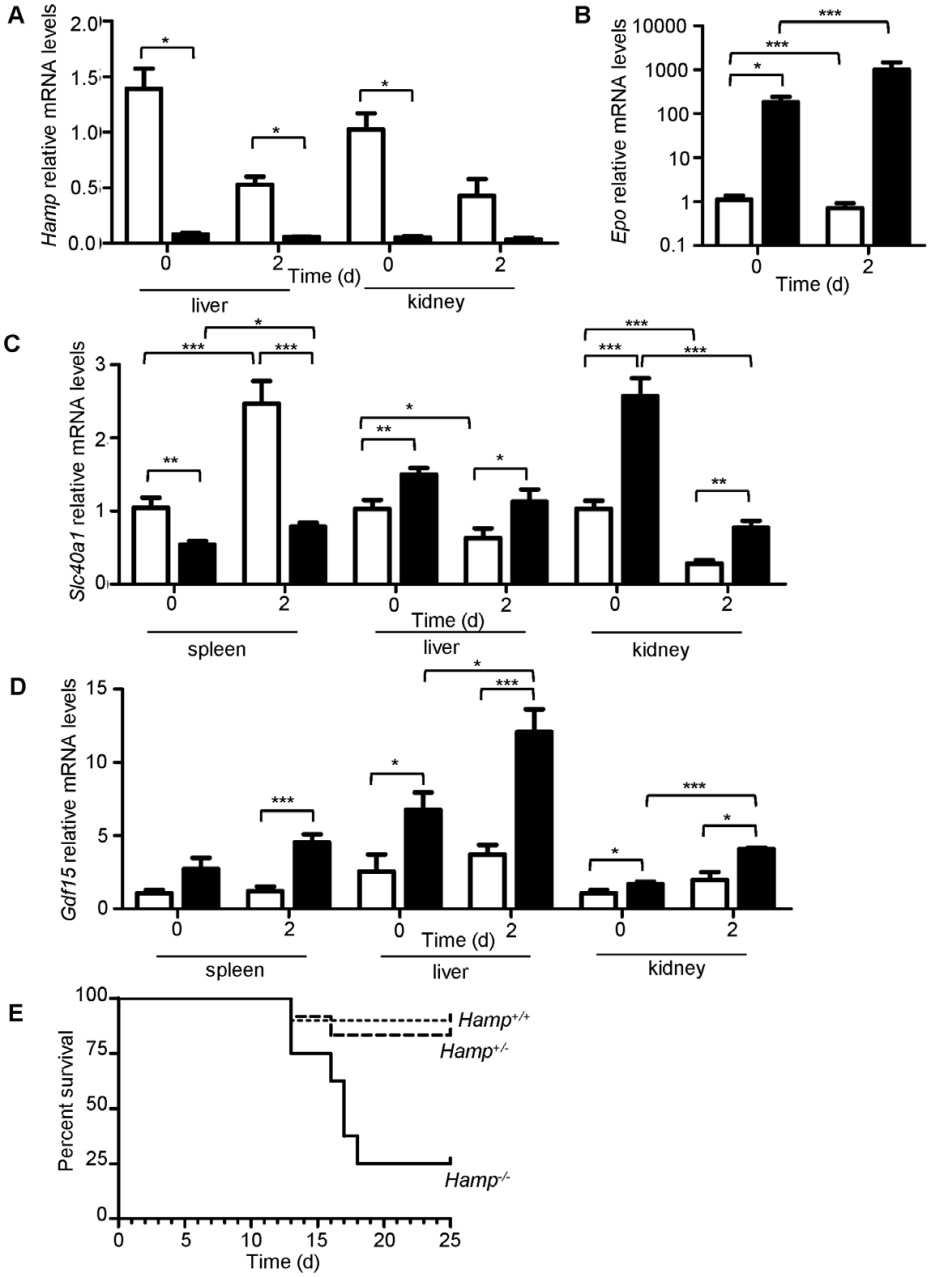
Tissue expression of genes involved in iron metabolism in 7 week old *Ank1^+/+^* wild type and *Ank1^Ity16/Ity16^* mutant mice. Real-time PCR expression of liver *Hamp* (A), kidney *Epo* (B), spleen, liver and kidney *Slc40a1* (C), and spleen and liver *Gdf15* (D). The relative mRNA levels at day 0 and day 2 post infection are shown in *Ank1^+/+^* wild type (clear bar, n = 3) and *Ank1^Ity16/Ity16^* mutant (black bar, n = 3) mice. *Epo* mRNA levels are represented on a LOG_10_ scale. (E) Survival curves of *Hamp* knock out mice (129S6.B6*129S2-Hamp^tm1Svl^) infected with 8000 CFUs of *Salmonella* Typhimurium. Solid line represents homozygous *Hamp^−/−^* knock out (n = 8), dashed line represents heterozygous *Hamp^+/−^* (n = 12), and dotted line represents homozygous wildtype *Hamp^+/+^* alleles (n = 10). Log-Rank (Mantel-Cox) for *Hamp^+/+^* and *Hamp^−/−^* p = 0.0071. An *represents a P-value of less than 0.05; ** represents a P-value of less than 0.001; *** represents a P-value of less than 0.0001.

**Figure 7:**
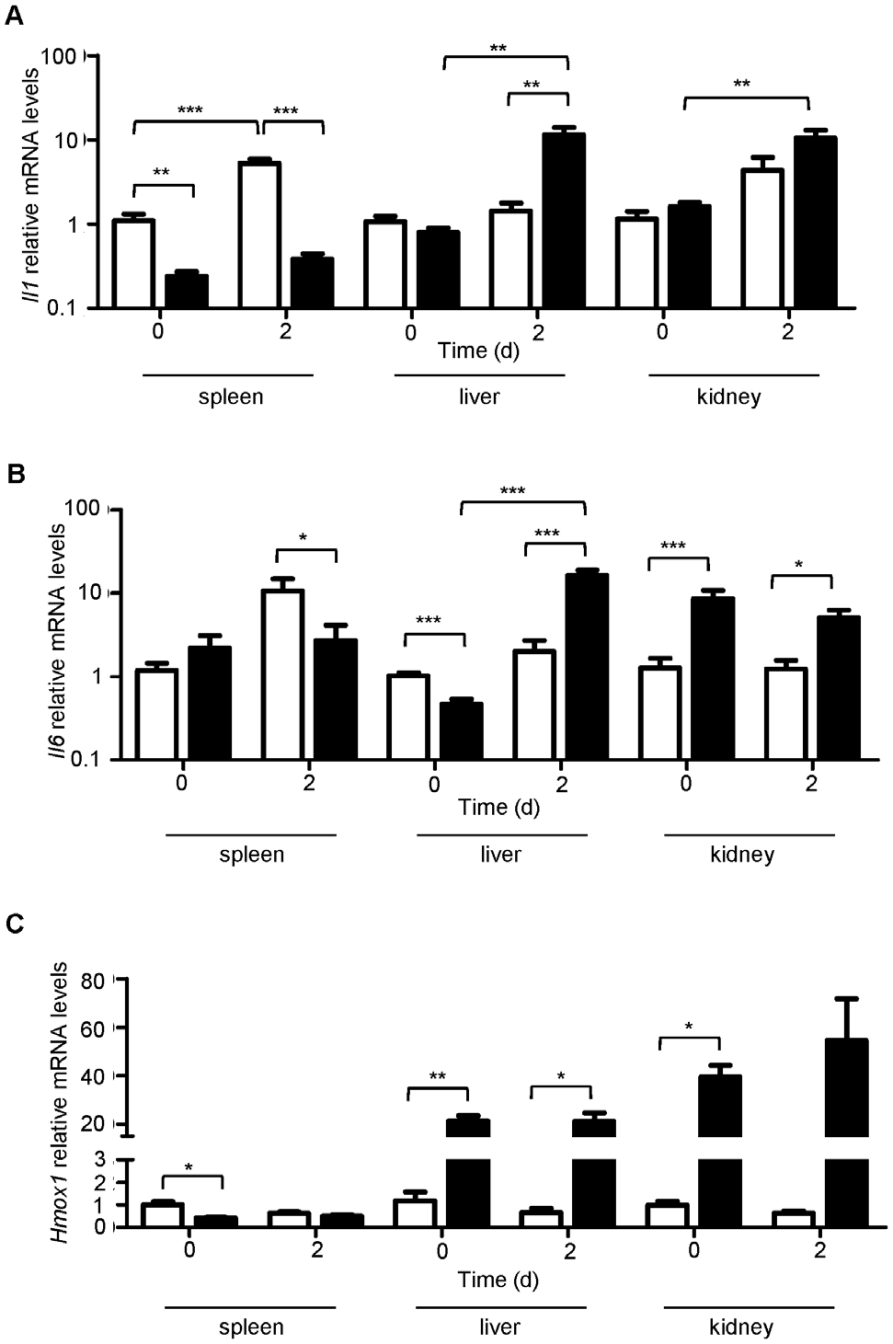
Cytokine profiles in 7 week-old *Ank1^+/+^* wild type and *Ank1^Ity16/Ity16^* mutant mice during infection with *Salmonella* Typhimurium. Relative spleen, liver and kidney mRNA levels are shown for *Il1* (A),*Il6* (B), and *Hmox1* (C) at day 0 and day 2 post infection. *Ank1^+/+^* wild type mice (n = 3) are represented by clear bar and *Ank1^Ity16/Ity16^* mutant mice (n = 3) by a black bar. All values are compared to wild type mRNA levels at day 0 and presented on a LOG_10_ scale. An * represents a P-value of less than 0.05; ** represents a P-value of less than 0.001; *** represents a P-value of less than 0.0001.

Because of the severe iron overload present in the liver and kidney of *Ank1^Ity16/ity16^* mice, the expression of key genes involved in iron metabolism including hepcidin (*Hamp*), ferroportin (*Slc40a1*) and heme oxygenase 1 (*Hmox1*) and regulators of *Hamp* expression (*Epo, Gdf15, Il1 and Il6*) were investigated (Fig. 6 and 7). The liver is a major site of iron storage and in clinical conditions of iron overload, the liver become a major site of iron deposition. The kidneys have been shown also to play a role in iron homeostasis and to express both HAMP and SLC40A1 [28], [29], [30]. In *Ank1^Ity16/Ity16^* mice, the liver and the kidneys are major site for *Salmonella* replication and growth during infection. The expression of *Hamp* in both uninfected and infected *Ity16* mutant mice was significantly downregulated in the liver and kidneys compared to wild type littermates (Fig. 6A). On the other hand, the liver and kidney expression of the membrane iron exporter, *Slc40a1* and *Hmox1* was significantly higher in *Ank1^Ity16/Ity16^* mice at day 0 and day 2 post infection compared to *Ank1^+/+^* (Fig. 6C and 7C). In the spleen of *Ank1^Ity16/ity16^* mutant mice, *Slc40a1* and *Hmox1* mRNA levels were significantly lower compared to wild-type littermates (Fig. 6C and 7C). In *Ank1^Ity16/Ity16^* mice, low *Hamp* expression had only a modest impact on the expression of liver *Slc40a1* both at the mRNA (Fig. 6C) and protein levels (data not shown). We investigated the expression of two inhibitory erythroid regulators of *Hamp* expression, erythropoietin (*Epo*) (Fig. 6B) and growth differentiation factor 15 (*Gdf15*) (Fig. 6D). These analyses revealed a major significant increase in the expression of both *Epo* and *Gdf15* only in *Ank1^Ity16/ity16^* mutant mice that could explain, at least in part, the low *Hamp* expression levels observed in these mice. During infection, the levels of *Gdf15* were significantly more elevated in *Ank1^Ity16/ity16^* mutant mice compared to wild type littermates (Fig. 6D). In addition, cytokine mRNA levels (*Il1*, and *Il6*) known to be upregulated during *Salmonella* infection and to impact on *Hamp* transcription were measured (Fig. 7A–B). The spleen of *Ank1^Ity16/ity16^* mutant mice appeared to be underresponsive to infection-induced cytokines as measured by very little or no induction of *Il1* and *Il6* (Fig. 7A–B). In the liver and the kidneys, the situation is different and *Ank1^Ity16/ity16^* mice showed marked increases in *Il1* (liver and kidneys) and *Il6* (liver) mRNA expression during infection (Fig. 7A). In these two tissues, high levels of *Il1* and *Il6* cytokines did not promote *Hamp* transcription in *Ank1^Ity16/ity16^* mutant. To further demonstrate the importance of *Hamp* during *Salmonella* infection, we did challenge *Hamp* knockout mice with *Salmonella* Typhimurium (Fig. 6E). We showed that mice deficient for *Hamp* (*Hamp^−/−^*) were significantly more susceptible to infection than mice carrying one (*Hamp^+/−^*) or two (*Hamp^+/+^*) (Mantel-Cox test P = 0.0079) wild type allele at *Hamp*. These results confirm the importance of *Hamp* during acute systemic model of *Salmonella* Typhimurium infection.

### *Salmonella* Susceptibility in *Ank1^+/Ity16^* Heterozygous Mice is Associated with Low Levels of *Hamp* and Iron Accumulation in Tissues

**Figure 8:**
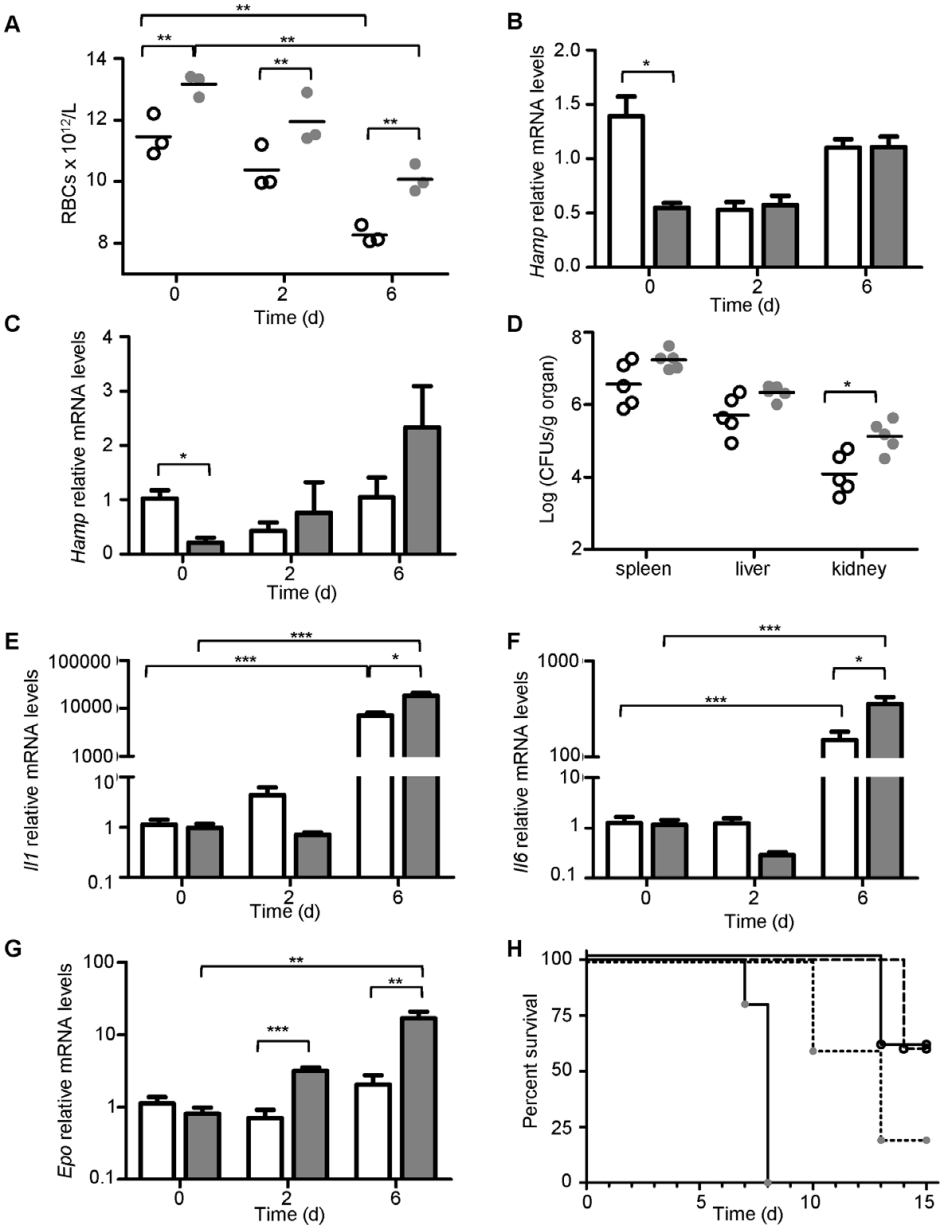
Characterization of *Ank1^+/Ity16^* heterozygous mice. RBC counts (A) and bacterial burden in spleen, liver and kidney (D) were measured in 7 week old *Ank1^+/+^* wild type and *Ank1^+/Ity16^* heterozygous mice at day 0 (prior infection), day 2 and day 6 post infection. Wild type *Ank1^+/+^* mice are represented by clear circles and *Ank1^+/Ity16^* heterozygous mice by gray circles. Relative kidney mRNA levels are shown for *Hamp* (C), *Il1* (E), *IL6* (F) and *Epo* (G) at day 0, day 2 and day 6 post infection. (B) Relative liver *Hamp* levels at day 0, day 2 and day 6 post infection. Wild type *Ank1^+/+^* mice are represented by clear bars (n = 3 for day 0 and day 2; n = 4 for day 6) and *Ank1^+/Ity16^* heterozygous mice by gray bars (n = 3 for day 0 and day 2; n = 4 for day 6). (H) Survival curves of *Ank1^+/+^* wild type (n = 5; open circle) and *Ank1^+/Ity16^* heterozygous (n = 5; gray circle) mice with (n = 5; dashed or dotted line) or without (n = 5; solid line) HAMP treatment infected with 5000 CFUs of *Salmonella* Typhimurium. Log-Rank (Mantel-Cox) for *Ank1^+/Ity16^* heterozygous with and without HAMP treatment p = 0.008. The experiment was repeated twice. An * represents a P-value of less than 0.05; ** represents a P-value of less than 0.001; *** represents a P-value of less than 0.0001.

*Ank1* ***^+/Ity16^*** heterozygous mice present an intermediate phenotype with respect to susceptibility to infection (survival) when compared to *Ank1^+/+^* and *Ank1^Ity16/Ity16^* littermates (Fig. 1E). Clinically, *Ank1* ***^+/Ity16^*** mice did not present any sign of anemia (Table 1) or splenomegaly (Fig. 2C). However, we did observe a small but significant increase in the number of RBCs (Fig. 8A). In addition, the *Ank1^+/Ity16^* mice presented moderate extramedullary hematopoiesis in the spleen (Fig. S1) and lower levels of *Hamp* expression in the liver (Fig. 8B) and kidneys (Fig. 8C) that was paralleled by increased expression of *Gdf15* (data not shown).

Early during infection (day 2), *Ank1* ***^+/Ity16^*** mice behaved as wild type littermates for most subphenotypes we have measured including different blood parameters (Table 1) and organ CFUs (Fig. 4). To understand the pathophysiology of the underlying susceptibility of *Ank1^+/Ity16^* mice, we followed the animals for a longer period of time during infection. At day 6 post infection when the animals become clinically diseased, *Ank1* ***^+/Ity16^*** mice presented consistent increase in bacterial load in the spleen, liver and kidney with a significant difference detected in the kidney (Fig. 8D). At day 6 post infection, iron pigment deposition was detected in the liver and kidneys of *Ank1^+/Ity16^* mice and not in wild type littermates (Fig. S3). The increase in kidney bacterial load together with iron deposition following infection clearly induced a local inflammatory response that is reflected by higher expression of renal *Il1* (Fig. 8E) and *Il6* (Fig. 8F) mRNA levels by a factor of 2.5 fold compared to wild-type controls. During infection, both wild-type and *Ank1* ***^+/Ity16^*** heterozygous mice developed a mild anemia although the *Ank1* ***^+/Ity16^*** heterozygous mice still presented higher RBC counts compared to control mice (Fig. 8A). The higher red cell mass present in *Ank1* ***^+/Ity16^*** heterozygous mice during infection could be explained by increased *Epo* expression by the kidneys (Fig. 8G). During infection, liver *Hamp* expression levels were not significantly modulated in both wild-type mice and *Ank1* ***^+/Ity16^*** heterozygous mice (Fig. 8B) although we did observe a modest but significant increase in kidney *Hamp* expression in *Ank1* ***^+/Ity16^*** mice later during infection (Fig. 8C). To test the involvement of Hamp in the susceptibility phenotype of *Ank1* ***^+/Ity16^*** heterozygous mice, we treated the mice with HAMP peptide. *Ank1* ***^+/Ity16^*** heterozygous mice treated with HAMP peptide showed significant survival benefits (Log Rank (Mantel-Cox) test p = 0.008) compared to heterozygous mice treated with PBS confirming the importance of HAMP in the host response to *Salmonella* infection (Fig. 8H).

## Discussion

We report here the identification of a novel mutation (*Ity16*) in the gene *Ank1* identified in an ENU recessive screen for susceptibility to typhoid-like disease in mice. The mutation consists in a nonsense mutation (p.Gln1357Ter) located in the spectrin binding domain of *Ank1*. ANK1 protein was not detected in RBC ghosts suggesting that *Ank1^Ity16^* is a null allele although we could not exclude the possibility that low levels of other ANK1 isoforms lacking the N-terminal region may remain undetected. Mice carrying the homozygous allele for *Ank1^Ity16^* present clinicopathological features of human hereditary spherocytosis (HS) which is the most common cause of inherited chronic hemolysis in Europe and North America with a prevalence in population of 1 in 2000 [31], [32]. Clinical manifestations of the human disease range from mild subclinical to severe life-threatening [12]. HS is characterized by spherocytic erythrocytes, splenomegaly, hyperbilirubinemia, thrombosis, leukocytosis and cardiac hypertrophy [19] and is predominantly caused by mutations in one of the erythroid membrane cytoskeleton components including ANK1, Band 3 (SLC4A1), α-spectrin (SPNA), β-spectrin (SPNB), and protein 4.2 (EBP4.2) [12]. Mutations in the *ANK1* gene accounts for about 50% of genetically defined cases of HS and most cases of HS associated with *ANK1* mutations [19](>80%) show a dominant mode of inheritance [12]. In HS and in mice lacking ANK1, the consequence of the ineffective erythropoiesis, extramedullary hematopoiesis and retention of abnormal RBC is splenomegaly and tissue iron overload leading to oxidative damage and cardiac failure (reviewed in [33]).

In addition to the mutation described in the current paper, five mutations within the *Ank1* locus have been reported (Fig. 3B). Two of them were spontaneous recessive mutations (*Ank1^nb^* and *Ank1^pale^*), two additional one were identified in dominant ENU screens for blood cell phenotypes (*Ank1^RBC2^* and *Ank1^E924X^*) and the last one, in a dominant screen for resistance to malaria in SJL/J mice (*Ank1^MRI23420^*) [13], [14], [15], [16], [17]. Mice carrying the *Ity16*, *nb*, *RBC2*, *E924X* or MRI23420 allele at *Ank1* present characteristic clinicopathological features of HS including severe anemia, reticulocytosis, splenomegaly with complete effacement of the normal splenic architecture, multiorgan iron overload and low body weight. Embryonic and neonatal lethality is observed and may vary according to the genetic background and the position of the mutation ([14], [15] and current paper). *Ank1^Ity16/Iy16^* mutants are on a mixed DBA/2J X 129/S1 background and most homozygous are viable (18%; the expected ratio is 25%) and survive to at least 6 months of age. In mutant *Ank1* mice where there is 100% neonatal mortality, the death was associated with severe clinical signs of jaundice due to massive hemolysis [17].

The severity of anemia in *Ank1^Ity16/Ity16^* mutant mice and other ENU-induced *Ank1* mutants (*Ank1^RBC2^* and *Ank1^E924X^*) was similar with hematocrit levels varying between 22–27%. Reticulocytosis was present in all mutants and significantly different from control mice. *Ank1^Ity16/Ity16^* mutant mice exhibit extensive iron accumulation in several organs including the kidneys, a pathological observation that was not reported in other *Ank1* mutants [13], [14], [15], [16]. A normal iron balance in the host is required for adequate innate and adaptive immune responses. This is well illustrated by the observation that both iron overload and iron depletion impaired the host immune response in humans and in animal models of infections [5], [6], [9], [34]. Iron overload is known to influence the course of infection by favoring microbial replication and also by affecting antimicrobial immune effector mechanisms. Iron overload has several consequences on the immune system including decreased capacity of macrophage to phagocytose, reduced neutrophil migration, modifications of T-cell subsets, suppression of the complement system, and increased oxidative stress leading to tissue damage [35]. Additionally, acute iron depletion in mice increased their susceptibility to *Salmonella* infection because of impaired NADPH-dependent respiratory burst activity [36]. In the current study, we present several evidences showing that iron overload is an important mechanism in influencing bacterial load including the concomitant presence of iron overload and high bacterial burden in liver and kidneys and the observation that *Salmonella* strain deficient in iron uptake grows better in the iron rich environment of *Ank1^Ity16/Ity16^* liver. We do also show that in heterozygous *Ank1^+/Ity16^* mice, higher bacterial load is detected only in tissues where iron accumulation is detected. These results clearly show the importance of iron overload in susceptibility to *Salmonella* infection in *Ank1^Ity16/Ity16^* and *Ank1^+/Ity16^* mice.

In *Ank1* mutant mice and HS patients, the normal life span of erythrocytes in the peripheral blood is substantially shortened and retention of abnormal erythrocytes by the spleen was shown to be the dominant mechanism for their reduced life-span [12], [14]. Hemolysis of erythrocytes results in the liberation of heme which induces the expression of *Hmox1.* HMOX1 catalyzes the degradation of heme to carbon monoxide, biliverdin that is converted to bilirubin and ferrous iron [37]. In *Ank1^Ity16/Ity16^* mutant mice, we observed high serum bilirubin levels and iron overload in the liver and the kidneys but not in the spleen. High *Hmox1* mRNA levels was observed in the liver and the kidneys but not in the spleen of mutant mice suggesting that the iron overload present in the liver and kidneys resulted from degradation of heme by *Hmox1*. Low levels of *Hmox1* in the spleen of mutant mice are most likely a consequence of splenic macrophage depletion (data not shown) and could explain the observation that there is no iron deposition in the spleen of mutant mice and less bacterial growth considering that hemophagocytic macrophages may provide a survival niche for *Salmonella* [38]. Of particular interest, increased levels of *Hmox1* has been recently shown to impair resistance to *Salmonella* infection in a context of hemolysis through the suppression of the oxidative burst capacity of neutrophils [39], suggesting that high *Hmox1* levels in *Ank1^Ity16/Ity16^* mutant mice may contribute to their susceptibility.

It has been shown that the kidney plays an important role in iron metabolism. It has also been reported that a significant amount of serum iron is filtered by the glomeruli and is reabsorbed [40]. In *Ank1^Ity16/Ity16^* mutant mice, the excessive iron load in the kidneys most likely results from the high rate of hemoglobin filtration and reabsorption by renal tubular cells. Iron deposition in glomeruli, and proximal and distal tubules of the kidney has been observed in chronic experimental hemosiderosis [41]. With aging, *Ank1^Ity16/Ity16^* mutants develop a more severe nephropathy compared to heterozygous and wild type animals and are more susceptible to infection as measured by increased in bacterial proliferation upon infection. The higher degree of iron accumulation in liver and kidneys in older animals is most likely responsible for these observations.

Another hallmark of ineffective erythropoiesis in *Ank1^Ity16/Ity16^* mutant mice is an increase in *Epo* and *Gdf15* levels associated with suppression of *Hamp* expression. *Hamp* expression is known to be regulated by intestinal iron absorption, iron recycling by macrophages and iron mobilization from hepatic stores by inhibiting iron export through its binding to *Slc40a1* causing its internalization [42]. In *Ank1^Ity16/Ity16^* mice, low *Hamp* expression had a modest impact on the expression of *Slc40a1* in the liver. The synthesis of *Hamp* is also upregulated in hepatocytes by inflammatory cytokines and inhibited by anemia, hypoxia and erythropoietic activity [43], [44]. As a consequence of anemia and in response to tissue hypoxia, increased expression of *Epo* is detected in the kidneys. Despite these changes to compensate for erythrocyte demand, erythropoisesis is not efficient and led to massive expansion of the erythroid compartment. GDF15 is a member of the TGFβ superfamily that was shown to negatively regulate the expression of *Hamp in vitro* [45], [46]. In fact, upregulation of GDF15 and suppression of *HAMP* have been observed in β-Thalassemia and it is thought that erythroid expansion influences the regulation of HAMP expression through systemic release of GDF15 from erythroblasts [30], [47]. Low levels of *Hamp* in response to ineffective erythropoiesis, as observed in *Ank1^Ity16/Ity16^* mutant mice, clearly exacerbate extramedullary erythropoiesis, tissue iron deposition and splenomegaly. During infection, liver *Hamp* expression levels remain suppressed and *Gdf15*, *Il1* and *Il6* expression increased significantly in *Ank1^Ity16/Ity16^* mutant mice compared to wild type littermates. *Gdf15* has been shown to be highly expressed in macrophages stimulated with LPS and to be modulated by several cytokines including IL-1 [48] and by intracellular iron depletion in vitro [49]. The high *Gdf15* expression observed during infection in *Ank1^Ity16/Ity16^* mutant mice could be explained by the progression of anemia and expansion of the erythroid compartment, by the high expression of cytokines induced by infection with *Salmonella* or as a consequence of intracellular iron deprivation due to low levels of *Hamp* expression.

*Salmonella* susceptibility in *Ank1^Ity16/Ity16^* differs from that observed in the iron overload disorder, hereditary hemochromoatosis (HFE). *Hfe*-deficient mice do not lack *Hamp*, present excessive accumulation of iron in the liver and improve resistance to *Salmonella* infection [50]. In *Hfe*-deficient mice, high lipocalin-2 (*Lcn2*) levels were observed in the liver and the spleen prior infection and increased resistance to infection in these mice was associated with higher induction of *Lcn2* expression that was shown to reduce the availability of iron for *Salmonella* within macrophages [50]. In *Ank1^Ity16/Ity16^* mutant mice, the spleen and liver expression of *Lcn2* did not differ from that observed in littermate controls prior infection however we did observe a significant increased in the spleen and liver expression of *Lcn2* during infection (data not shown). There were no significant differences between genotypes in the spleen in contrast levels of *Lcn2* were significantly higher (by a factor of 1.5 LOG) in the liver of *Ank1^Ity16/Ity16^* mutant mice compared to littermate controls and parallel the high bacterial loads detected in this organ. Altogether, these data suggest that high levels of *Lcn2* expression in a context of low *Hamp* expression do not protect *Ank1^Ity16/Ity16^* mutant mice from systemic *Salmonella* infection.

Of particular interest was the intermediate susceptibility of *Ank1^+/Ity16^* mice to infection with *Salmonella* Typhimurium. In heterozygous mice, there is only one functional copy of *Ank1* which may be limiting to confer a normal structure to the RBC membrane and cause a partial loss of membrane surface. Although the half-life of RBC appears not to be affected in *Ank1^+/Ity16^* mice, small increase in osmotic fragility has been reported which may lead to more RBC destruction by the spleen [14]. In fact, we did observe an increase in extramedullary erythropoiesis in the spleen of *Ank1^+/Ity16^* mice although the mice were not anemic. The phenotype observed in *Ank1^+/Ity16^* mice may correspond to the human mild form of HS where patients have compensated hemolysis without anemia. As observed in uninfected *Ank1^Ity16/Ity16^* homozygous mice, liver and kidneys *Hamp* levels were low compared to controls and levels of *Gdf15* were increased. *Hamp* is mainly produced by the liver but it has been found also to be highly expressed in the apical pole of epithelial cells of distal tubules and collecting ducts of the kidney [29], [51]. In humans, diminished serum hepcidin concentration has been observed in human erythroid pathologies where there is no anemia but an increased in red cell mass as seen in primary polycythemia [52]. In *Ank1^+/Ity16^* mice, the *Salmonella* susceptible phenotype appears to be expressed predominantly in the kidney where we found significant accumulation of iron and bacteria. Low levels of *Hamp* expression in *Ank1^+/Ity16^* heterozygous mice at the time of infection appears to contribute to iron accumulation in the kidney and liver and consequently favor bacterial growth. The central role of *Hamp* in the host response to *Salmonella* infection was validated using mice deficient for *Hamp*. These mice are not anemic and present an important iron overload secondary to low level of *Hamp* a phenotype similar to the one observed in heterozygous mice [21] and are highly susceptible to *Salmonella* infection (current paper). Finally, direct evidence of the role of *Hamp* in the host response to *Salmonella* infection was obtained by treating *Ank1^+/Ity16^* mice with hepcidin. Hepcidin treatment did not improve resistance of *Ank1^Ity6/Ity16^* mutant mice where iron accumulation in tissues is massive however it was clearly beneficial in *Ank1^+/Ity16^* heterozygous mice. We do not know at the moment the exact mechanism of how hepcidin is improving resistance to infection in *Ank1^+/Ity16^* heterozygous mice. Induction of hepcidin through transgenesis in mouse models of hemochromatosis and β-Thalassemia was shown to alter the pattern of cellular iron accumulation and limit iron overload [53], [54]. In addition, administration of an hepcidin agonist in Hamp^−/−^ mice caused a partial redistribution of iron from the liver to the spleen [55]. An antimicrobial effect of hepcidin could be also considered although the impact of hepcidin on bacterial growth has been shown only in vitro [56].

Overall, the current study shows that the suppression of *Hamp* expression and iron overload contribute to the susceptibility of *Ank1^Ity16/Ity16^* mutant and *Ank1^+/Ity16^* heterozygous mice to *Salmonella* infection. This emphasizes the importance of iron metabolism and a role for *Hamp* in susceptibility to systemic *Salmonella* infection.

## Supporting Information

Figure S1 **Histopathologic examination of the spleen, liver and kidney of 7 week old** ***ANK1^+/+^*** **wild type,** ***ANK1^+/Ity16^*** **heterozygous, and** ***ANK1^Ity16/Ity16^*** **mutant mice before infection (day 0).** H&E stain of uninfected spleen of wild type (A), heterozygous (E), and Ity16 mutants (I). Prussian blue stain of uninfected spleen, liver and kidney, respectively, of wild type (B,C,D), heterozygous (F,G,H), and Ity16 mutants (J,K,L). All pictures are taken at 200×magnification. RP = red pulp, WP = white pulp.
(PDF)

Figure S2 **Progression of lesions in kidney and liver 2 days after** ***Salmonella*** **infection of** ***ANK1^+/+^*** **wild type,** ***ANK1^+/Ity16^*** **heterozygous, and** ***ANK1^Ity16/Ity16^*** **mutant mice aged 7 and 24 weeks using hematoxylin & eosin staining.** H&E stain of uninfected kidney of wild type (A,B), heterozygous (E,F), and Ity16 mutants (I,J) at 7 and 24 weeks of age respectively. H&E stain of day 2 post infection liver of wild type (C,D), heterozygous (G,H), and Ity16 mutants (K,L) at 7 and 24 weeks of age respectively. All pictures taken at 200×magnification.
(PDF)

Figure S3 **Prussian blue staining of liver and kidney of 7 week old** ***ANK1^+/+^*** **wild type and** ***ANK1^+/Ity16^*** **heterozygous mice at day 2 and day 6 post infection with** ***Salmonella*** **Typhimurium.** Prussian blue stain of day 2 post infection liver in wild type (A) and heterozygous (D) mice at 200×magnification. Prussian blue stain of day 6 post infection liver of wild type (B) and heterozygous (E) mice at 200×magnification. Prussian blue stain of day 6 post infection kidney of wild type (C) and heterozygous (F) mice at 1000×magnification.
(PDF)

Table S1 **Primer sequences used for quantitative PCR.** Two housekeeping genes: *Tbp* (TATA box binding protein) and *Hprt* (hypoxanthine guanine phosphoribosyl transferase) were used. Hepcidin (*Hamp*), ferroportin (*Slc40a1*), interleukin 6 (*Il6)*, inteleukin 1 (*Il1*), interferon gamma (*Ifng*), erythropoietin (*Epo*), heme oxygenase 1 (*Hmox1*) and growth differentiation factor 15 (*Gdf15*).
(XLSX)
