# Supplementary material for: Suppression of Hepcidin Expression and Iron Overload Mediate Salmonella Susceptibility in Ankyrin 1 ENU-Induced Mutant
Source: PLoS One. 2013 Feb 4;8(2):e55331. doi: 10.1371/journal.pone.0055331 (PMC3563626; doi:10.1371/journal.pone.0055331)

Supplemental Figure 1: Histopathologic examination of the spleen, liver and kidney of 7 week old *Ank1*$^{+/+}$ wildtype, *Ank1*$^{+/Ity16}$ heterozygous, and *Ank1*$^{Ity16/Ity16}$ mutant mice before infection (day 0)

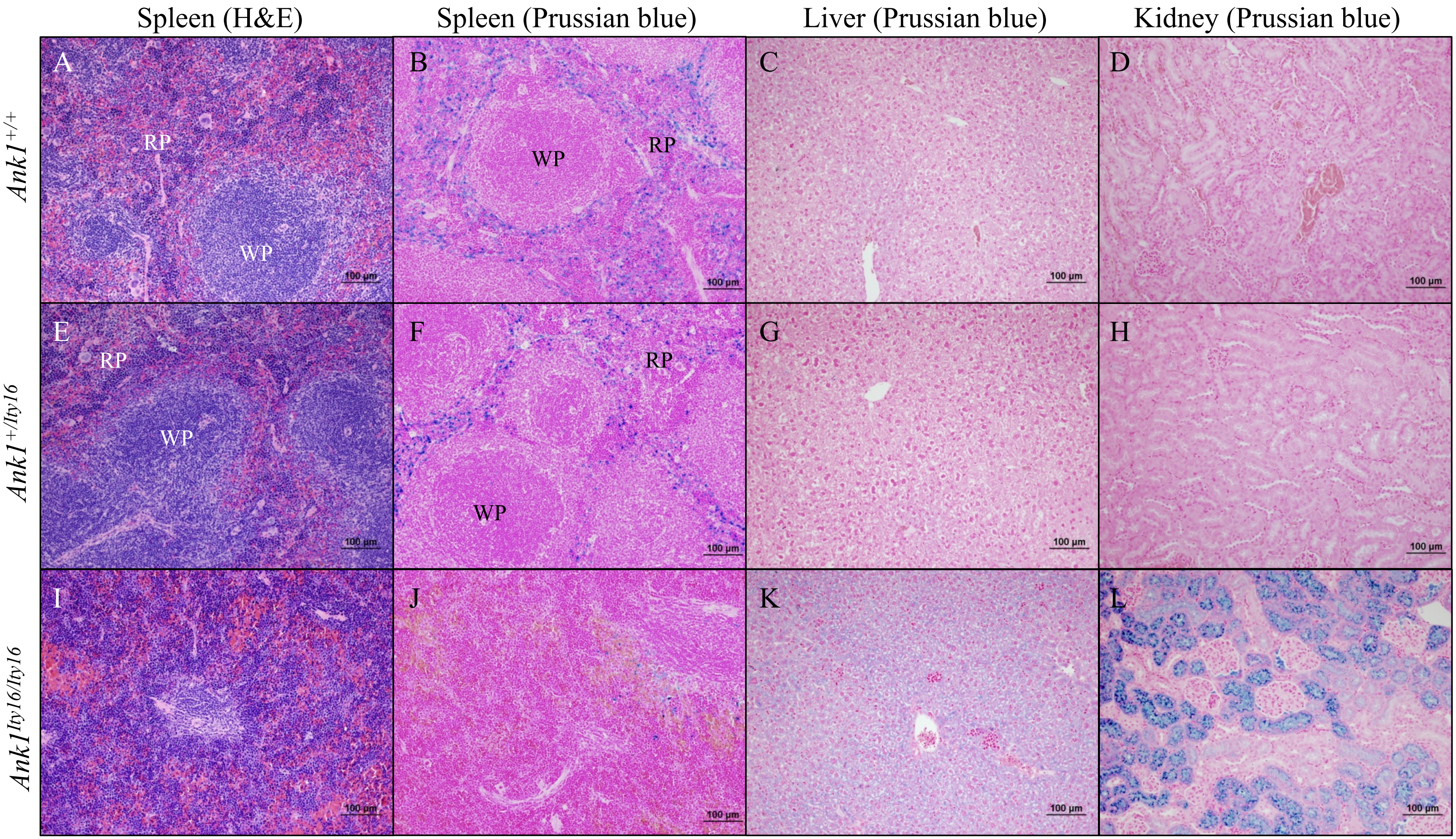

Supplement: Figure S1 — Histopathologic examination of the spleen, liver and kidney of 7 week old ANK1+/+ wild type, ANK1+/Ity16 heterozygous, and ANK1Ity16/Ity16 mutant mice before infection (day 0). H&E stain of uninfected spleen of wild type (A), heterozygous (E), and Ity16 mutants (I). Prussian blue stain of uninfected spleen, liver and kidney, respectively, of wild type (B,C,D), heterozygous (F,G,H), and Ity16 mutants (J,K,L). All pictures are taken at 200×magnification. RP = red pulp, WP = white pulp. (PDF) [file pone.0055331.s001.pdf]
