# Supplementary material for: Suppression of Hepcidin Expression and Iron Overload Mediate Salmonella Susceptibility in Ankyrin 1 ENU-Induced Mutant
Source: PLoS One. 2013 Feb 4;8(2):e55331. doi: 10.1371/journal.pone.0055331 (PMC3563626; doi:10.1371/journal.pone.0055331)

Supplemental Figure 2: Progression of lesions in kidney and liver 2 days after *Salmonella* infection of *Ank1*$^{+/+}$ wildtype, *Ank1*$^{+/Ity16}$ heterozygous, and *Ank1*$^{Ity16/Ity16}$ mutant mice aged 7 and 24 weeks using hematoxylin & eosin staining.

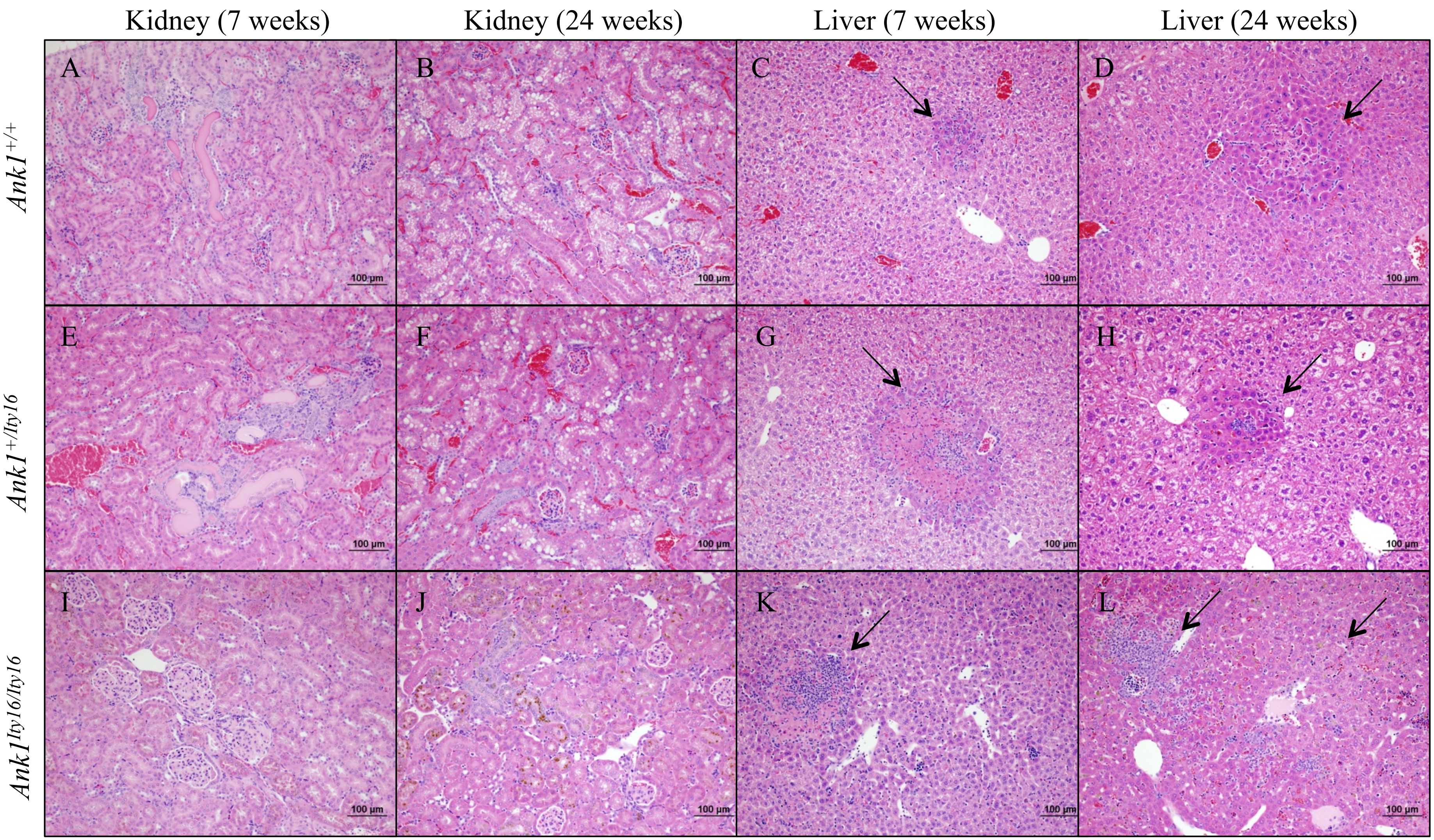

Supplement: Figure S2 — Progression of lesions in kidney and liver 2 days after Salmonella infection of ANK1+/+ wild type, ANK1+/Ity16 heterozygous, and ANK1Ity16/Ity16 mutant mice aged 7 and 24 weeks using hematoxylin & eosin staining. H&E stain of uninfected kidney of wild type (A,B), heterozygous (E,F), and Ity16 mutants (I,J) at 7 and 24 weeks of age respectively. H&E stain of day 2 post infection liver of wild type (C,D), heterozygous (G,H), and Ity16 mutants (K,L) at 7 and 24 weeks of age respectively. All pictures taken at 200×magnification. (PDF) [file pone.0055331.s002.pdf]
