# Supplementary material for: Suppression of Hepcidin Expression and Iron Overload Mediate Salmonella Susceptibility in Ankyrin 1 ENU-Induced Mutant
Source: PLoS One. 2013 Feb 4;8(2):e55331. doi: 10.1371/journal.pone.0055331 (PMC3563626; doi:10.1371/journal.pone.0055331)

Supplemental Figure 3: Prussian blue staining of liver and kidney of 7 week old *Ank1*$^{+/+}$ wild type, and *Ank1*$^{+/lty16}$ heterozygous mice at day 2 and day 6 post infection with *Salmonella* Typhimurium

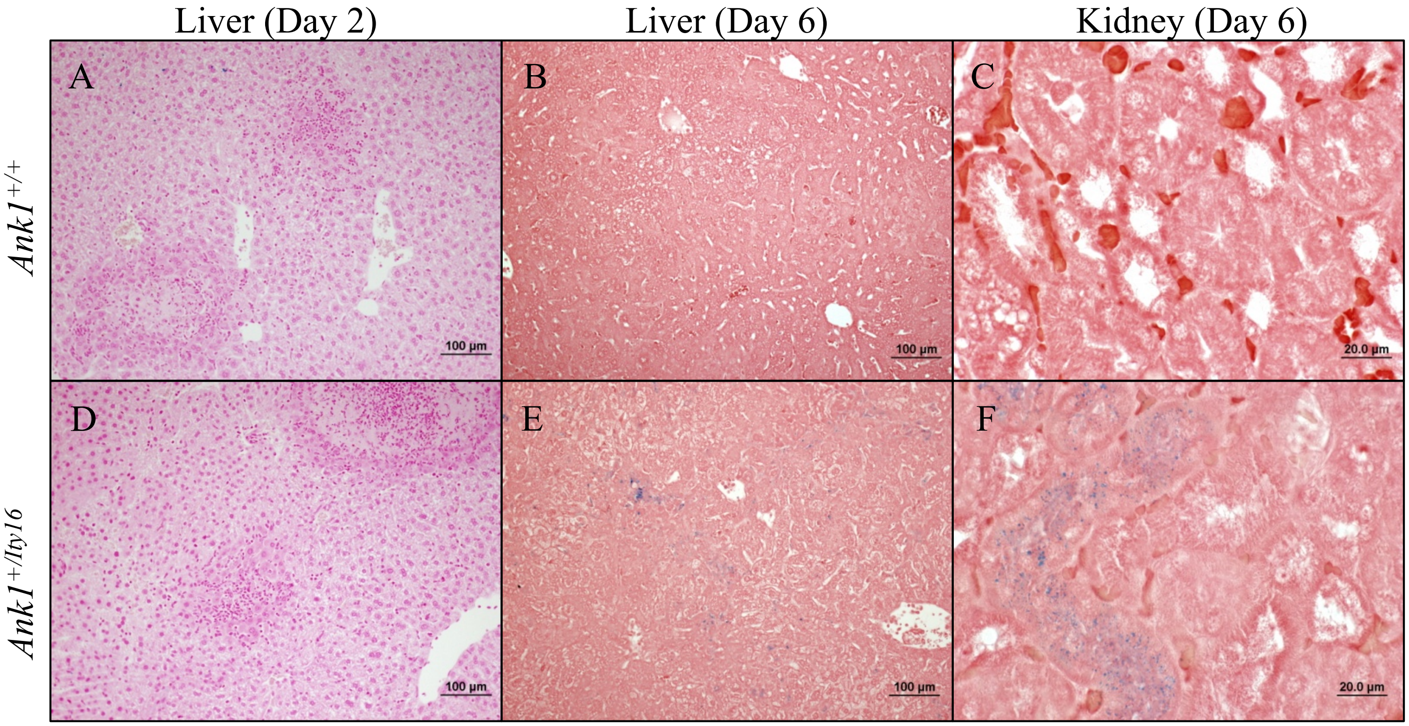

Supplement: Figure S3 — Prussian blue staining of liver and kidney of 7 week old ANK1+/+ wild type and ANK1+/Ity16 heterozygous mice at day 2 and day 6 post infection with Salmonella Typhimurium. Prussian blue stain of day 2 post infection liver in wild type (A) and heterozygous (D) mice at 200×magnification. Prussian blue stain of day 6 post infection liver of wild type (B) and heterozygous (E) mice at 200×magnification. Prussian blue stain of day 6 post infection kidney of wild type (C) and heterozygous (F) mice at 1000×magnification. (PDF) [file pone.0055331.s003.pdf]
